# Influencing cognitive performance via social interactions: a novel therapeutic approach for brain disorders based on neuroanatomical mapping?

**DOI:** 10.1038/s41380-022-01698-1

**Published:** 2022-07-20

**Authors:** Suzanne D. Lanooij, Ulrich L. M. Eisel, Wilhelmus H. I. M. Drinkenburg, Eddy A. van der Zee, Martien J. H. Kas

**Affiliations:** 1 Groningen Institute for Evolutionary Life Sciences (GELIFES), Neurobiology, University of Groningen, Nijenborgh 7, 9747 AG Groningen, The Netherlands; 2 Department of Neuroscience, Janssen Research & Development, a Division of Janssen Pharmaceutica NV, Turnhoutseweg 30, B-2340 Beerse, Belgium

**Keywords:** Neuroscience, Psychiatric disorders

## Abstract

Many psychiatric and neurological disorders present deficits in both the social and cognitive domain. In this perspectives article, we provide an overview and the potential of the existence of an extensive neurobiological substrate underlying the close relationship between these two domains. By mapping the rodent brain regions involved in the social and/or cognitive domain, we show that the vast majority of brain regions involved in the cognitive domain are also involved in the social domain. The identified neuroanatomical overlap has an evolutionary basis, as complex social behavior requires cognitive skills, and aligns with the reported functional interactions of processes underlying cognitive and social performance. Based on the neuroanatomical mapping, recent (pre-)clinical findings, and the evolutionary perspective, we emphasize that the social domain requires more focus as an important treatment target and/or biomarker, especially considering the presently limited treatment strategies for these disorders.

## Introduction

Social and cognitive deficits are shared symptoms among many psychiatric and neurological disorders. For example, social withdrawal is often one of the first behavioral indicators of schizophrenia, depression, and Alzheimer’s Disease (AD) [1]. Similarly, patients with one of these disorders can have problems with working memory, attention, and proper sensory processing [1]. Given the striking overlapping of symptoms in different neuropsychiatric disorders, we hypothesized that there is an underlying neurobiological substrate to explain the relationship between social and cognitive deficits. Therefore, we have mapped the neurobiological substrates of the domain of sociality (i.e., the tendency to associate in groups) and of cognition to assess the relationship between these two domains. Next, we reflected on how dysfunction in one domain can influence the other domain, and provided implications of this close relationship for therapeutic strategies for these disorders, focusing on AD as an example. While the current therapeutic strategies to treat AD symptoms are mainly focusing on the cognitive domain, we put forward support to additionally focus on the social domain to alleviate both social and cognitive deficits in AD patients.

To provide a neurobiological understanding of the brain substrates underlying the interaction between social and cognitive performance, here we mainly focus on rodent studies. In rodents, it is possible to examine and manipulate specific neuronal circuits and/or brain regions and couple those to social and cognitive performance to study causal relationships under well-controlled (environmental) conditions. In humans, research thus far has been dominated by assessing correlations between behavior and neuroimaging, rather than causality. In addition, the ability to alter rodents’ genetics has led to novel opportunities to create models for human neurological disorders [2]. Moreover, rodents express a vast variety of social behaviors and cognitive functions and their social and cognitive neural substrates show substantial evolutionary conservation [3]. While we will discuss some limitations in the translational validity of such models in the next section, these properties render rodents valuable models to study the interconnected neurobiology of social behavior and cognition [4].

### Overlapping neurobiological substrates of sociality and cognition

Sociality is a shared characteristic across species. The comparable social behaviors that have been observed in a wide range of species hint towards conserved biological mechanisms. Research in the field of social cognition has focused on identifying the so-called ‘social brain’, in order to obtain a framework for neuroscientists to understand the neural mechanisms of social behavior. Brothers and coworkers in 1990 proposed a network consisting of brain regions involved in specialized social cognition in primates (mostly neocortex), known as the Cognitive Social Brain (CSB) network [5]. The characterization of the CSB was mainly based on lesion studies, single-unit recordings, and neuroimaging techniques in primates. In a different manner, Newman’s Social Behavior Network (SBN) described the core of the social brain (mostly limbic forebrain and midbrain structures) involved in more primitive social functioning, which is present in all vertebrates [6]. The nodes of this network are bi-directionally connected, express sex steroid hormone receptors, and are implicated in the control of multiple forms of social behavior. The brain regions were identified using histological imaging methods and genomic and transcriptomic assessments of mRNA expression in the rodent brain. The CSB and SBN differ in their characterization approach, but both aspire to characterize the social brain. Therefore, Prounis et al. (2020) argue that the SBN and CSB are actually two parts of the same larger social brain and should thus collectively be considered as one [7]. Furthermore, as the rewarding nature of social behavior is a driving force for social contact, O’Connell & Hofmann proposed the “social decision-making network” (SDMN), which includes the SBN and the mesolimbic reward system [8]. Evidence for the role of this network comes from neurochemical, tract-tracing, developmental, and functional lesion/stimulation studies. The SDMN is highly conserved across vertebrate taxa and constitutes an important foundation for the understanding of the neural circuits underlying social behavior.

**Fig. 1:**
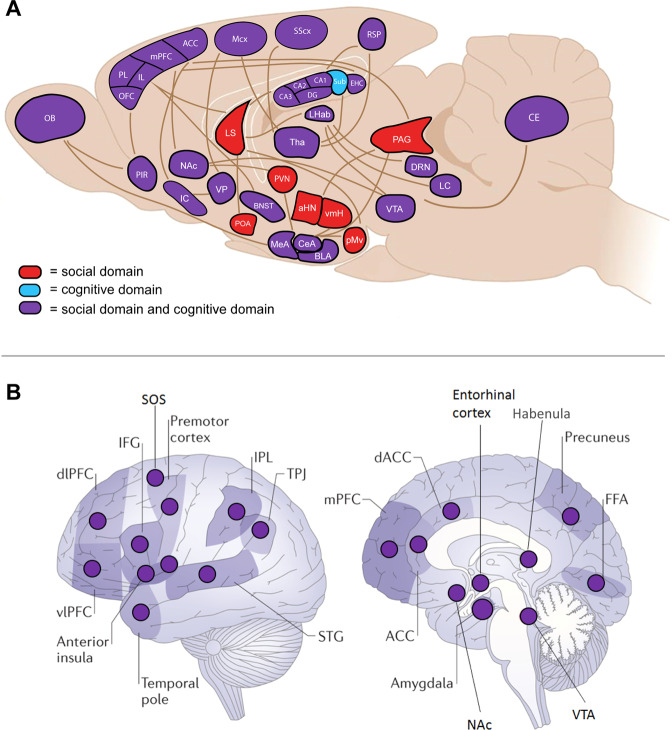
Brain regions involved in the social and/or cognitive domain. The depicted brain regions are involved in the social domain (red), cognitive domain (blue), or in both domains (purple). **A** A sagittal illustration of the rodent brain showing our identified brain regions and their involvement in the social and/or cognitive domain. Several connections between brain areas are drawn to illustrate that brain regions act in a network. Image was adapted with permission from Ike et al., *Neurosci Biobehav Rev*. (2020) [60]. **B** Human brain indicating brain regions relevant to schizophrenia, major depressive disorder, and/or Alzheimer’s Disease. All these brain regions are involved in both social and cognitive functioning. The image was adapted with permission from Porcelli et al., *Neurosci Biobehav Rev*. (2019) [36]. ACC anterior cingulate cortex, aHN anterior hypothalamic nucleus, BLA basolateral amygdala, BNST bed nucleus of the stria terminalis, CA cornu ammonis, CE cerebellum, CeA central amygdala, dlPFC dorsolateral prefrontal cortex, DRN dorsal raphe nucleus, EHC entorhinal cortex, FFA fusiform face area, IC insular cortex, IFG inferior frontal gyrus, IL infralimbic cortex, IPL inferior parietal lobule, LC locus coeruleus, LHab lateral habenula, LS lateral septum, Mcx motor cortex, meA medial amygdala, mPFC medial prefrontal cortex, NAc nucleus accumbens, OB olfactory bulb, OFC orbitofrontal cortex, PAG periaqueductal gray, PIR piriform cortex, PL prelimbic cortex, pMv ventral premammilary nucleus, POA medial preoptic area, PVN paraventricular nucleus, RSP retrosplenial area, SOS superior orbital sulcus, SScx somato-sensory cortex, STG superior temporal gyrus, Sub subiculum, Tha thalamus, TPJ temporo-parietal junction, vlPFC ventrolateral prefrontal cortex, VMH ventral medial hypothalamus, VP ventral pallidum, VTA ventral tegmental area.

In order to map the neural substrate for the connection between social functioning and cognition, we used the three original brain networks (CSB, SBN, and SDMN) as a starting point. Next, based on additional and more recent literature on the connections between social and cognitive functioning and the rodent brain, we added additional brain regions to our overview (Fig. 1 and Supplementary Table 1). The literature was identified using PubMed search terms related to social functioning (e.g., “social behavior”, “sociability”, “sociality”) or cognitive functioning (e.g., “cognitive performance”, “memory”). The included studies used behavioral, neurophysiological, histological, and molecular readouts. Brain regions that facilitate social behavior and all functionality that is required to enable it, including internal processes (e.g., social cue processing, social memory formation) as well as observable actions (e.g., aggression, social withdrawal), were considered to be involved in the social domain. We mapped the brain regions that are part of the cognitive domain using the definition of Shettleworth on animal cognition: “all ways in which animals take in information about the world through the senses, process, retain and decide to act on it” [9]. For each identified brain region, its involvement in the other domain, being either social or cognitive functioning, was subsequently checked by performing a literature search using the earlier described PubMed search terms. The brain regions in our overview can be involved at different stages/levels of social and/or cognitive functioning (e.g., perception, processing, decision-making, learning, memory, and execution) and may have a direct or more indirect role in a process. We used the references (and the references therein) listed in Supplementary Table 1 to categorize a brain region as being part of the social and/or cognitive domain. Besides involvement in social or cognitive functioning as defined above, no other inclusion or exclusion criteria were applied. This has led to a broader overview of brain regions involved in social behavior, compared to the original social networks. Notably, the brain structures from our overview that are involved in the social domain but not in the cognitive domain, are hypothalamic structures and cerebral nuclei. These regions belong to an older part of the brain with respect to brain evolution, and accordingly, these structures are involved in more basic social behaviors necessary for survival, such as mating and huddling for thermoregulation [10]. More complex social behaviors (e.g., social decision-making) require cognitive skills and are generated by structures of the limbic system and neocortex [8]. Indeed, most of those structures are implicated as being part of both the social and the cognitive domain.

Furthermore, considerable functional overlap is identified in brain regions involved in the perception or processing of environmental stimuli (e.g., the piriform cortex, thalamus, somatosensory cortex), which is keeping in with the notion that a stimulus can be social or non-social. Likewise, proper functioning of the motor cortex is essential to properly execute social and non-social behaviors. Of note is that the degree of overlap of the social and cognitive domain within a brain region depends on the subdivisions made within that region; a structure may be considered to be involved in both domains but subdividing this region into smaller regions such as layers or subnuclei may reveal there is less or even no overlap at this smaller scale. For example, within the orbitofrontal cortex, Jennings et al identified distinct cell populations that selectively responded to either social stimuli or caloric rewards [11]. This emphasizes the importance of considering the network environment in which the cell populations function, which will be addressed in more detail in the section below on network structures.

Clearly, the overlap of brain regions that contribute to both social functioning and cognitive functioning is extensive. Evidently, the previously mentioned overlap in symptoms seen across neuropsychiatric disorders thus has a neurobiological basis. Only for the subiculum no evidence of the involvement of this specific region in the social domain was found in the literature to date. However, considering its involvement in hippocampal-cortical interaction, a role for this structure in social functioning is likely [12].

Importantly, the identified brain regions are part of several network structures and thus are not independent regulators. Detailed descriptions of the interaction between brain regions in different networks have been described in a variety of review papers [3, 8, 13, 14]. Functional networks require functional synaptic activity as well as functional anatomical connections between brain regions, which mainly constitute myelinated fibers (e.g., white matter). White matter integrity is therefore also of great importance for social and cognitive functioning and has received increased attention over the past few years, also in relation to neurological and psychiatric brain disorders [15, 16]. Moreover, specific white matter structures have been shown to be implicated in social cognition. For example, microstructural integrity of the forceps minor is linked to social network diversity in humans, and microstructural changes in this area were found in socially isolated mice [17, 18].

Furthermore, over the past years, advancing insights into the function of different brain regions has elucidated additional components of the social- and cognitive- brain networks. Indeed, the focus of attention that brain structures receive, and the context in which they are studied, contribute to the anticipated value of the necessity of regularly updating this kind of overviews. Currently still understudied brain regions may as well be involved in the social and/or cognitive domain. We are aware that our overview might be limited by reflecting the present, incomplete state of knowledge, but we believe that this nonetheless offers a valuable framework for the understanding of the interplay between the social and cognitive domain in neurological and psychiatric disorders.

### An evolutionary link between social and cognitive functioning

Sociality is an adaptive function to cope with ecological challenges that ultimately can contribute to an individual’s reproductive fitness. For example, group living lowers the susceptibility to predations, helps to acquire resources (food, shelter, mates), and can reduce costs for thermoregulation [19]. Basic social interactions depend little on cognitive skills and are mostly generated by older brain structures in terms of brain evolution (hypothalamus and cerebral nuclei). Yet, group living is challenging and involves more complex social behavior, including recognition memory and calculating the consequences of specific types of behavior, which requires cognitive processes involving cortical structures. This increased need for social complexity solving (i.e., social intelligence) requires an increase in computing power and thus brain size.

More complex cognition is observed in several different species (e.g., birds and non-human primates) and is thought to have evolved through a process of convergent evolution [20, 21]. Within primates, brain expansion is thought to have coevolved with sociality [22, 23]. Indeed, several theories consider that sociality has contributed to the evolution of intelligence (high-order cognition). For example, Humphrey’s influential social intelligence hypothesis argues that social complexity and social competition are the driving forces for the evolution of primate intelligence. He deliberates that social skills have been applied to solve practical problems (high-order intelligence) and thus hypothesizes that there should be a positive correlation across species between ‘social complexity’ and ‘individual intelligence’ [24]. In line with this hypothesis, Dunbar showed that the size of the neocortex relative to the rest of the body is a strong predictor of group size, much stronger than diet and habitat. According to his social brain hypothesis, social complexity is the driving force for the increased brain size of primates [25].

The social brain hypothesis was originally based on observations in primates, but has been generalized (in an adapted form) to all vertebrate taxa to explain brain evolution [26]. In non-primate mammals, brain size correlates with the social complexity of mating systems. Especially animals that pair-bond (yearly or life-long) have a larger neocortex resulting in more complex brain computing power [27]. For a wide range of mammals, dealing with social challenges rather than environmental challenges, appear the biggest intellectual problem to be faced [28].

The neuroanatomical overlap of the social and the cognitive domain can thus be explained from an evolutionary point of view. Neural circuits act as generalist rather than specialist and thus new functions can arise from the same neural circuit upon small changes (e.g., altering connections) [29]. The evolution of the brain has enabled new behaviors, and more complex social behavior could thus have been constructed from existing neural circuits for nonsocial behaviors [23]. However, it has been postulated that human brain-specific cognitive advancements may have caused vulnerability of the brain for neuropsychiatric disorders [16, 30–32].

### Species-specific aspects of behavior and the translation to humans

Despite the evolutionary conserved similarities in the social brain of humans and rodents, the rodent brain differs from the human brain, particularly in the size and functioning of the cortex. Especially the human prefrontal cortex is more advanced and the existence of a convincing rodent homologue is still under debate [33, 34]. The neuroanatomical differences likely relate to the behavioral differences between humans and rodents. Certain aspects of social behavior, like social interaction (e.g., language), social organization (e.g., hierarchy building), social bonds (e.g., pair-bonding) are expressed in a species-specific manner. Despite these differences in the expression and appearance of social behavior between species, they serve the same evolutionary relevant purpose, namely reproduction and reproductive fitness of offspring [35].

### Implications for treatment strategies of psychiatric and neurological disorders

The identification of a neurobiological substrate contributing to the close relationship between the social and cognitive domain offers new insights into treatment strategies for psychiatric and neurological disorders. Porcelli et al. (2019) mapped the human brain regions associated with social functioning that are implicated in AD, schizophrenia, and/or major depressive disorder [36]. Their overview shows the overlap in pathways associated with these disorders, as well as the relationship between these disorders and the social brain. Interestingly, all the indicated human brain regions as indicated by Porcelli et al. (2019) are also involved in cognitive processes (Fig. 1B and Supplementary Table 2), which supports that our hypothesis of neurobiological overlap of social and cognitive functioning is applicable to humans as well, and illustrates the relevance of our framework for neurological and psychiatric disorders. In the following section, we will focus on the implications of the relationship between social and cognitive functioning on treatments for AD as an example.

Traditionally, cognitive problems are the main focus for symptomatic treatment strategies for neurodegenerative disorders, including AD. Cognitive performance, therefore, is a key read-out in pharmacological and non-pharmacological clinical trials for AD. However, besides the cognitive impairments, 80% of the patients with mild cognitive impairment or dementia will at some point in their disease progression display behavioral and psychological symptoms (BPSD) including mood disturbances and altered social behavior [37]. Conversely, human association studies have shown that poor social engagement increases dementia risk, whereas good social engagement exerts a mildly protective effect [38]. Moreover, many brain regions impacted by AD pathology are involved in both the social and cognitive domain, including the prefrontal cortex, anterior cingulate cortex, anterior insula, amygdala, nucleus accumbens, superior orbital sulcus, and ventral tegmental area (Fig. 1) [36]. Furthermore, the human Default Mode Network (DMN) has been implicated in cognitive and social functioning and alterations in its functional connectivity are associated with loneliness and social functioning in several psychiatric disorders and AD [39, 40]. Indeed, the DMN encompasses for a large part the same brain areas as the earlier described social brain networks. Taken together, these findings emphasize the relevance of both domains in the pathology and treatment of AD and support our hypothesis that the social domain constitutes an important target in addition to the cognitive domain.

**Fig. 2:**
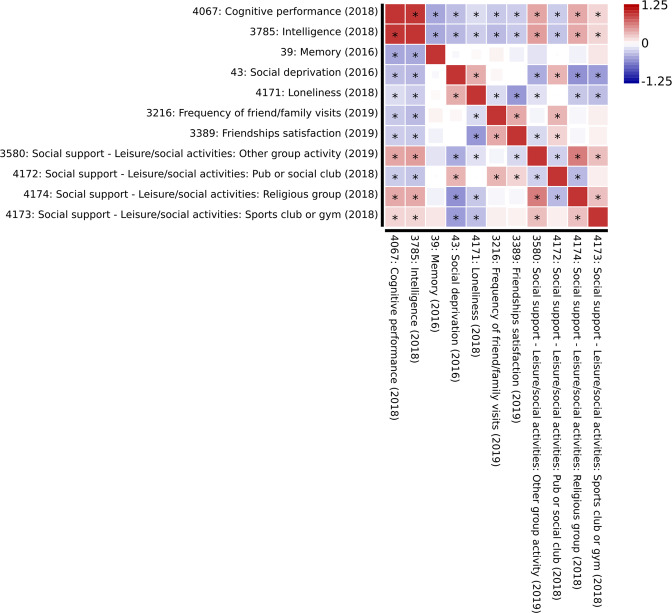
Genetic correlation between social and cognitive functioning. These results were obtained from the GWAS Atlas (https://atlas.ctglab.nl/) [43]. The selected traits come from UK Biobank studies with >100.000 participants per study. Significant genetic correlations after Bonferroni correction (<0.05) are labeled with an asterisk. The colour indicates the direction and strength of the genetic correlation.

Interestingly, overlap between social functioning and cognitive functioning can also be found at the genetic level. Many genetic loci have been identified related to cognitive functioning [41]. Sociability also has a genetic basis [42]. Using the GWAS Atlas (https://atlas.ctglab.nl/ [43]), we identified a strong genetic correlation between several cognitive traits and social traits (Fig. 2). These findings further underscore the relationship between these two domains.

The causal relationship between the social and cognitive domain has been investigated in rodent studies. Many studies report increased performance on cognitive tasks in socially housed rodents compared to socially deprived rodents [44–48]. Socially isolated animals have repeatedly been shown to display altered behavior, including increased anxiety, impaired memory, reduced synaptic plasticity, and altered expression of several neuropeptides and neurochemicals [45, 48–50]. Moreover, the social environment can have an effect on AD pathophysiology in mice [46]. Also, socially isolated animals often display increased social motivation, implying a “social need” to be fulfilled [51].

The social environment of the rodent home cage can affect similar mechanisms that play a role in psychiatric disorders. Moreover, in line with findings of human studies, an enriched social environment has been shown to exert positive effects on the brain and behavior of rodents and can mitigate cognitive decline caused by neurodegeneration [52–54]. This is in line with human findings showing that greater social health correlates with better cognitive performance and reduced risk to develop AD [38, 55–58].

Several studies have shown additional benefits of social enrichment over physical and/or cognitive enrichment [38, 47, 56]. Given the framework provided here, it would be an important next step to perform large randomized clinical trials specifically targeting the social domain to slow down the progression of AD. Possibly, the social intervention procedure requires thorough optimization and in humans possibly personalization, taking into account factors like personality traits and disease stage [59]. Based on the shared neurobiological substrates of the social domain and cognitive domain, we propose that adding more focus on the social domain may contribute to improving both social and cognitive functioning of AD patients.

Social interventions are also promising treatment strategies for other neuropsychiatric disorders that share symptoms and have overlap in affected brain regions and connections [36, 40]. A better understanding of the intricate relationship between symptoms, brain function, and social interventions may furthermore help identifying optimal (combination) therapies for individual patients and patient subgroups.

## Conclusion

The overlapping social and cognitive symptoms in psychiatric and neurological disorders have a neuroanatomical basis, which can be explained from an evolutionary perspective. Considering the extensive overlap between the social and the cognitive domain and evidence from animal studies for a functional interplay between these domains, targeting the social domain in treatment strategies for psychiatric and neurological disorders holds promise in alleviating both social and cognitive symptoms. Including the social domain in the present limited options for brain disease therapies offers novel and valuable opportunities for patients with a high medical need.

## Supplementary information

Supplementary table 1

Supplementary table 2
