# Supplementary material for: Influencing cognitive performance via social interactions: a novel therapeutic approach for brain disorders based on neuroanatomical mapping?
Source: Mol Psychiatry. 2022 Jul 20;28(1):28–33. doi: 10.1038/s41380-022-01698-1 (PMC9812764; doi:10.1038/s41380-022-01698-1)
Supplement: Supplementary file 1 — Supplementary table 1 [file 41380_2022_1698_MOESM1_ESM.doc]

**Supplementary Table 1**

References (and the references therein) used to identify the involvement of rodent brain regions in the social and or cognitive domain. The main functions used to categorize the brain regions are indicated. Less well-established functions of a brain region are indicated in bold and the method and behavioral task used in the reference to identify this function is specified between brackets.

| **Brain region** | **Subregion** | **Functions in the social domain** | **Functions in the cognitive domain** |
| --- | --- | --- | --- |
| Amygdala |  | Processing social cues, social behavior [1,2] | Decision-making, memory [3] |
|  | medial amygdala | (social) odor processing, reproductive behavior, aggression  [1,4,5] | Sensory processing, Odor processing [1,5] |
|  | basolateral amygdala | Social behavior [1,5,6] | Fear conditioning [5,6] |
|  | central amygdala | Social behavior [1,4] | **Fear conditioning** (pharmacology) [7] |
| Bed nucleus stria terminalis |  | Aggression, reproductive behavior [1,8] | Fear conditioning [1,8] |
| Cerebellum |  | **Social cognition, sociability** (pharmacology, behavior: *Three chamber task)* [9,10] | Cognitive processing, executive functioning [9,10] |
| Dorsal raphe nucleus |  | Social behavior [4], **loneliness** (optogenetics; *three chamber, place preference task, social isolation*) [11] | Motivation, attention, reward, decision-making, learning and memory [4] |
| Entorhinal cortex |  | **Social memory** (optogenetics; *three chamber task*) [12] | Memory [13] |
| Frontal cortex | Anterior cingulate cortex | Social recognition memory [6], aggression [14] | Decision-making [15], motivation, sensory processing, memory [6,16] |
|  | mPFC | Social behavior, social recognition [4,6] | Memory, decision-making, behavioral inhibition [6,16] |
|  | Prelimbic cortex | **Social behavior** (*in vivo* electrophysiology: *social interaction test*) [17] | Fear response, reward behavior [16,18] |
|  | Infralimbic cortex | **Social behavior** (*in vivo* electrophysiology: *social interaction test*) [17] | Habit behavior, Fear behavior [16,18] |
|  | Orbitofrontal cortex | **Social behavior** (optogenetics: *social interaction tests*) [19] | Reversal learning, reward processing [16] |
| Hippocampus | CA1 | Social memory [6,20] | Learning and memory [6,21] |
|  | CA2 | Social memory [4,22] | (Social) memory,  [4,22,23] |
|  | CA3 | **Social memory** (behavior: *social recognition, social choice test*) [6,24] | Learning and memory [6,21] |
|  | DG | **Social memory** (optogenetics, pharmacology: *Three chamber task, direct social interaction test*) [12,25] | Learning and memory, pattern separation [21] |
| Hypothalamus | Preoptic area | Aggression, reproductive behavior, parental care [1] |  |
|  | Anterior hypothalamus | Aggression, maternal behavior [1] |  |
|  | Ventromedial hypothalamus | Reproductive behavior |  |
|  | Paraventricular nucleus | Social behavior and bonding  [26,27] |  |
|  | Ventral premammilary nucleus | Reproduction [28], maternal aggression [29] |  |
| Insular cortex |  | Social response, **empathy** (pharmacology: *writhing test*) [30] social decision-making [1,31,32] | Social-decision making [1,31,32] |
| Lateral habenula |  | Reproductive behavior [4,33], **Social information processing** (pharmacology: *social interaction test*) [34] | Reward learning, cognitive flexibility, goal-directed actions [4,33,35] |
| Lateral septum |  | Reproductive behavior, sociability, aggression [1,36] |  |
| Locus coeruleus |  | **Social stress** (*in vivo* electrophysiology: *resident-intruder test*), [37,38] **sociability** (behavior: *social interaction test*) [39] | Memory [40,41] |
| Motor cortex |  | Execution of social behavior [42] | Execution of cognitive tasks [42] |
| Olfactory bulb |  | Social odor perception, social recognition  [4,43] | Odor perception  [4,43] |
| Periaqueductal gray |  | Reproduction, aggression, vocal communication [1,4] |  |
| Piriform cortex |  | Social odor perception [43] | Odor perception [43] |
| Retrosplenial area |  | Experience of emotions [44] and **social isolation** (*pharmacology, immunohistochemistry*) [45] | Memory [46,47] |
| Somatosensory cortex |  | Sensory processing [48] | Sensory processing [48] |
| Striatum |  | Social reward [1,49] | Learning [1,49] |
|  | Nucleus accumbens | Social reward [1,50] , social memory [51] | Motivation, reward learning, decision-making [1,49,51] |
|  | Ventral pallidum | Social affiliation [1,52] | Motor output behavior, reward processing [1,52] |
| Subiculum |  |  | Memory [53] |
| Thalamus |  | Sensory processing, social interaction [54,55] | Sensory processing, Memory, cognitive flexibility [55] |
| Ventral tegmental area |  | Reproductive behavior, parental behavior [1,4] | Motivation, reward [1,56] |

**References for Supplementary Table 1**

1. O’Connell LA, Hofmann HA. The Vertebrate Mesolimbic Reward System and Social Behavior Network : A Comparative Synthesis. J Comp Neurol. 2011;3639:3599–639.

2. Bickart KC, Dickerson BC, Feldman L. The amygdala as a hub in brain networks that support social life. Neuropsychologia [Internet]. Elsevier; 2014;63:235–48. Available from: http://dx.doi.org/10.1016/j.neuropsychologia.2014.08.013

3. Gupta R, Koscik T, Bechara A, Tranel D. The amygdala and decision making. Neuropsychologia. 2011;49:760–6.

4. Ko J. Neuroanatomical substrates of rodent social behavior: The medial prefrontal cortex and its projection patterns. Front Neural Circuits. 2017;11:1–16.

5. Gangopadhyay P, Chawla M, Dal Monte O, Chang SWC. Prefrontal–amygdala circuits in social decision-making. Nat Neurosci [Internet]. Springer US; 2021;24:5–18. Available from: http://dx.doi.org/10.1038/s41593-020-00738-9

6. Tanimizu T, Kenney JW, Okano E, Kadoma K, Frankland PW, Kida X. Functional Connectivity of Multiple Brain Regions Required for the Consolidation of Social Recognition Memory. J Neurosci Res. 2017;37:4103–16.

7. Wilensky AE, Schafe GE, Kristensen MP, LeDoux JE. Rethinking the fear circuit: The central nucleus of the amygdala is required for the acquisition, consolidation, and expression of pavlovian fear conditioning. J Neurosci. 2006;26:12387–96.

8. Miles OW, Maren S. Role of the bed nucleus of the stria terminalis in PTSD: Insights from preclinical models. Front Behav Neurosci. 2019;13:1–14.

9. Badura A, Verpeut JL, Metzger JW, Pereira TD, Pisano TJ, Deverett B, et al. Normal cognitive and social development require posterior cerebellar activity. Elife. 2018;7:1–36.

10. Van Overwalle F, Manto M, Cattaneo Z, Clausi S, Ferrari C, Gabrieli JDE, et al. Consensus Paper: Cerebellum and Social Cognition. Cerebellum. The Cerebellum; 2020.

11. Matthews GA, Nieh EH, Vander Weele CM, Halbert SA, Pradhan R V., Yosafat AS, et al. Dorsal Raphe Dopamine Neurons Represent the Experience of Social Isolation. Cell [Internet]. The Authors; 2016;164:617–31. Available from: http://dx.doi.org/10.1016/j.cell.2015.12.040

12. Leung C, Cao F, Nguyen R, Joshi K, Aqrabawi AJ, Xia S, et al. Activation of Entorhinal Cortical Projections to the Dentate Gyrus Underlies Social Memory Retrieval. Cell Rep [Internet]. ElsevierCompany.; 2018;23:2379–91. Available from: https://doi.org/10.1016/j.celrep.2018.04.073

13. Morrissey MD, Takehara-Nishiuchi K. Diversity of mnemonic function within the entorhinal cortex: A meta-analysis of rodent behavioral studies. Neurobiol Learn Mem [Internet]. Elsevier Inc.; 2014;115:95–107. Available from: http://dx.doi.org/10.1016/j.nlm.2014.08.006

14. van Heukelum S, Tulva K, Geers FE, van Dulm S, Ruisch IH, Mill J, et al. A central role for anterior cingulate cortex in the control of pathological aggression. Curr Biol. 2021;31:2321-2333.e5.

15. Rudebeck PH, Walton ME, Smyth AN, Bannerman DM, Rushworth MFS. Separate neural pathways process different decision costs. Nat Neurosci. 2006;9:1161–8.

16. Dalley JW, Cardinal RN, Robbins TW. Prefrontal executive and cognitive functions in rodents: Neural and neurochemical substrates. Neurosci Biobehav Rev. 2004;28:771–84.

17. Minami C, Shimizu T, Mitani A. Neural activity in the prelimbic and infralimbic cortices of freely moving rats during social interaction: Effect of isolation rearing. PLoS One. 2017;12:1–22.

18. Mukherjee A, Caroni P. Infralimbic cortex is required for learning alternatives to prelimbic promoted associations through reciprocal connectivity. Nat Commun [Internet]. Springer US; 2018;9. Available from: http://dx.doi.org/10.1038/s41467-018-05318-x

19. Jennings JH, Kim CK, Marshel JH, Raffiee M, Ye L, Quirin S, et al. Interacting neural ensembles in orbitofrontal cortex for social and feeding behaviour. Nature. 2019;565:645–9.

20. Okuyama T, Kitamura T, Roy DS, Itohara S, Tonegawa S. Ventral CA1 neurons store social memory. Science (80- ). 2016;353:1536–41.

21. Zemla R, Basu J. Hippocampal function in rodents. Curr Opin Neurobiol. 2017;43:187–97.

22. Tzakis N, Holahan MR. Social Memory and the Role of the Hippocampal CA2 Region. Front Behav Neurosci. 2019;13:1–15.

23. Prasad Gabbita S, Johnson MF, Kobritz N, Eslami P, Poteshkina A, Varadarajan S, et al. Oral TNFα Modulation Alters Neutrophil Infiltration, Improves Cognition and Diminishes Tau and Amyloid Pathology in the 3xTgAD Mouse Model. PLoS One [Internet]. 2015 [cited 2018 Apr 23];10. Available from: https://www.ncbi.nlm.nih.gov/pmc/articles/PMC4593589/pdf/pone.0137305.pdf

24. Chiang MC, Huang AJY, Wintzer ME, Ohshima T, McHugh TJ. A role for CA3 in social recognition memory. Behav Brain Res [Internet]. Elsevier; 2018;354:22–30. Available from: https://doi.org/10.1016/j.bbr.2018.01.019

25. Cope EC, Waters RC, Diethorn EJ, Pagliai KA, Dias CG, Tsuda M, et al. Adult-Born Neurons in the Hippocampus Are Essential for Social Memory Maintenance. Eneuro. 2020;7:ENEURO.0182-20.2020.

26. Choleris E, Clipperton-Allen AE, Phan A, Kavaliers M. Neuroendocrinology of social information processing in rats and mice. Front Neuroendocrinol [Internet]. Elsevier Inc.; 2009;30:442–59. Available from: http://dx.doi.org/10.1016/j.yfrne.2009.05.003

27. Kirouac GJ. Placing the paraventricular nucleus of the thalamus within the brain circuits that control behavior. Neurosci Biobehav Rev [Internet]. Elsevier Ltd; 2015;56:315–29. Available from: http://dx.doi.org/10.1016/j.neubiorev.2015.08.005

28. Leshan RL, Pfaff DW. The hypothalamic ventral premammillary nucleus: A key site in leptin’s regulation of reproduction. J Chem Neuroanat [Internet]. Elsevier B.V.; 2014;61:239–47. Available from: http://dx.doi.org/10.1016/j.jchemneu.2014.08.008

29. Motta SC, Guimarães CC, Furigo IC, Sukikara MH, Baldo MVC, Lonstein JS, et al. Ventral premammillary nucleus as a critical sensory relay to the maternal aggression network. Proc Natl Acad Sci U S A. 2013;110:14438–43.

30. Zaniboni CR, Pelarin V, Baptista-de-Souza D, Canto-de-Souza A. Empathy for pain: Insula inactivation and systemic treatment with midazolam reverses the hyperalgesia induced by cohabitation with a pair in chronic pain condition. Front Behav Neurosci. 2018;12:1–10.

31. Rogers-Carter MM, Christianson JP. An insular view of the social decision-making network. Neurosci Biobehav Rev [Internet]. Elsevier; 2019;103:119–32. Available from: https://doi.org/10.1016/j.neubiorev.2019.06.005

32. Rogers-Carter MM, Varela JA, Gribbons KB, Pierce AF, McGoey MT, Ritchey M, et al. Insular Cortex Mediates Approach and Avoidance Responses to Social Affective Stimuli. Nat Neurosci. 2018.

33. Freudenmacher L, von Twickel A, Walkowiak W. The habenula as an evolutionary conserved link between basal ganglia, limbic, and sensory systems—A phylogenetic comparison based on anuran amphibians. J Comp Neurol. 2020;528:705–28.

34. van Kerkhof LWM, Damsteegt R, Trezza V, Voorn P, Vanderschuren JMJ. Functional intergrity of the habenula is necessary for social play behavior in rats. Eur J Neurosci. 2013;38.

35. Baker PM, Jhou T, Li B, Matsumoto M, Mizumori SJY, Stephenson-Jones M, et al. The lateral habenula circuitry: Reward processing and cognitive control. J Neurosci. 2016;36:11482–8.

36. Prounis GS, Ophir AG. Neuroscience and Biobehavioral Reviews One cranium , two brains not yet introduced : Distinct but complementary views of the social brain. Neurosci Biobehav Rev [Internet]. Elsevier; 2020;108:231–45. Available from: https://doi.org/10.1016/j.neubiorev.2019.11.011

37. Zitnik GA, Curtis AL, Wood SK, Arner J, Valentino RJ. Adolescent Social Stress Produces an Enduring Activation of the Rat Locus Coeruleus and Alters its Coherence with the Prefrontal Cortex. Neuropsychopharmacology. 2016;41:1376–85.

38. Chaijale NN, Snyder K, Arner J, Curtis AL, Valentino RJ. Repeated social stress increases reward salience and impairs encoding of prediction by rat locus coeruleus neurons. Neuropsychopharmacology [Internet]. Nature Publishing Group; 2015;40:513–23. Available from: http://dx.doi.org/10.1038/npp.2014.200

39. Jacobson L. Glucocorticoid receptor deletion from locus coeruleus norepinephrine neurons promotes depression-like social withdrawal in female but not male mice. Brain Res [Internet]. Elsevier B.V.; 2019;1710:82–91. Available from: https://doi.org/10.1016/j.brainres.2018.12.026

40. Mather M, Harley CW. The Locus Coeruleus: Essential for Maintaining Cognitive Function and the Aging Brain. Trends Cogn Sci [Internet]. Elsevier Ltd; 2016;20:214–26. Available from: http://dx.doi.org/10.1016/j.tics.2016.01.001

41. Sara SJ, Bouret S. Orienting and Reorienting: The Locus Coeruleus Mediates Cognition through Arousal. Neuron [Internet]. Elsevier Inc.; 2012;76:130–41. Available from: http://dx.doi.org/10.1016/j.neuron.2012.09.011

42. Schoen SA, Miller LJ. Motor Functioning and Adaptive Behavior. Adapt Behav Assess Syst. 2008. p. 245–66.

43. Wilson DA, Sullivan RM. Cortical Processing of Odor Objects. Neuron. 2011;72:506–19.

44. Ferris CF, Stolberg T, Kulkarni P, Murugavel M, Blanchard R, Caroline CD, et al. Imaging the neural circuitry and chemical control of aggressive motivation. BMC Neurosci. 2008;9:1–35.

45. Klimczak P, Rizzo A, Castillo-Gómez E, Perez-Rando M, Gramuntell Y, Beltran M, et al. Parvalbumin Interneurons and Perineuronal Nets in the Hippocampus and Retrosplenial Cortex of Adult Male Mice After Early Social Isolation Stress and Perinatal NMDA Receptor Antagonist Treatment. Front Synaptic Neurosci. 2021;13:1–13.

46. Miller AP, Vedder LC, Law ML, Smith DM. Cues, context, and long-term memory: The role of the retrosplenial cortex in spatial cognition. Front Hum Neurosci. 2014;8:1–15.

47. Milczarek MM, Vann SD, Sengpiel F. Spatial Memory Engram in the Mouse Retrosplenial Cortex. Curr Biol [Internet]. Elsevier Ltd.; 2018;28:1975-1980.e6. Available from: https://doi.org/10.1016/j.cub.2018.05.002

48. Hornix BE, Havekes R, Kas MJH. Multisensory cortical processing and dysfunction across the neuropsychiatric spectrum. Neurosci Biobehav Rev [Internet]. Elsevier; 2019;97:138–51. Available from: https://doi.org/10.1016/j.neubiorev.2018.02.010

49. Cox J, Witten IB. Striatal circuits for reward learning and decision-making. Nat Rev Neurosci. 2019;20:482–94.

50. Dölen G, Darvishzadeh A, Huang KW, Malenka RC. Social reward requires coordinated activity of nucleus accumbens oxytocin and serotonin. Nature. 2013;501:179–84.

51. Okuyama T. Social memory engram in the hippocampus. Neurosci Res [Internet]. Elsevier Ireland Ltd and Japan Neuroscience Society; 2018;129:17–23. Available from: http://dx.doi.org/10.1016/j.neures.2017.05.007

52. Smith KS, Tindell AJ, Aldridge JW, Berridge KC. Ventral pallidum roles in reward and motivation. Behav Brain Res. 2009;196:155–67.

53. O’Mara SM, Commins S, Anderson M, Gigg J. The subiculum: A review of form, physiology and function. Prog Neurobiol. 2001;64:129–55.

54. Ferguson BR, Gao W-J. Thalamic control of cognition and social behavior via regulation of GABAergic signaling and E/I balance in the medial prefrontal cortex. Physiol Behav. 2016;176:100–106.

55. Rikhye R V., Wimmer RD, Halassa MM. Toward an Integrative Theory of Thalamic Function. Annu Rev Neurosci. 2018;41:163–83.

56. Trutti AC, Mulder MJ, Hommel B, Forstmann BU. Functional neuroanatomical review of the ventral tegmental area. Neuroimage [Internet]. Elsevier Ltd; 2019;191:258–68. Available from: https://doi.org/10.1016/j.neuroimage.2019.01.062
