# Supplementary material for: Influencing cognitive performance via social interactions: a novel therapeutic approach for brain disorders based on neuroanatomical mapping?
Source: Mol Psychiatry. 2022 Jul 20;28(1):28–33. doi: 10.1038/s41380-022-01698-1 (PMC9812764; doi:10.1038/s41380-022-01698-1)
Supplement: Supplementary file 2 — Supplementary table 2 [file 41380_2022_1698_MOESM2_ESM.doc]

**Supplementary Table 2**

Overview of the human brain regions from Figure 1B and several main cognitive functions they are involved in.

| **Brain region** | **Function in cognitive domain** |
| --- | --- |
| Anterior cingulate cortex | Emotional control [1] |
| Anterior insula | Sensory processing, attention, decision-making [2] |
| Dorsal lateral Prefrontal cortex | Attention, working memory, planning [3] |
| Entorhinal cortex | Memory [4] |
| Fusiform face area | Facial response [5] |
| Habenula | Reward learning, decision-making [6] |
| Inferior frontal gyrus | Behavioral inhibition [7] |
| Inferior pariental lobule | Language, social cognition, attention [8] |
| Nucleus accumbens | Emotion, motivation, reward [9] |
| Precuneus | Consciousness, self-processing, episodic memory [10] |
| Premotor cortex | Preparation of movement [11] |
| Superior orbital sulcus | Contextual information processing [12] |
| Superior temporal gyrus | Auditory and language processing [13] |
| Temporo-parietal junction | Attention [14] |
| Ventral tegmental area | Reward, motivation, addiction [15] |
| Ventrolateral prefrontal cortex | Attention, response inhibition, language [3] |

**References Supplementary Table 2**

1. Stevens FL, Hurley RA, Taber KH. Anterior cingulate cortex: Unique role in cognition and emotion. J Neuropsychiatry Clin Neurosci. 2011;23:121–5.

2. Uddin LQ, Nomi JS, Hebert-Seropian B, Ghaziri J, Boucher O. Structure and function of the human insula. J Clin Neurophysiol. 2017;34:300–6.

3. Jones DT, Graff-Radford J. Executive Dysfunction and the Prefrontal Cortex. Contin Lifelong Learn Neurol. 2021;27:1586–601.

4. Schultz H, Sommer T, Peters J. The role of the human entorhinal cortex in a representational account of memory. Front Hum Neurosci. 2015;9:1–8.

5. Kanwisher N, Yovel G. The fusiform face area: A cortical region specialized for the perception of faces. Philos Trans R Soc B Biol Sci. 2006;361:2109–28.

6. Hikosaka O. The habenula: From stress evasion to value-based decision-making. Nat Rev Neurosci. Nature Publishing Group; 2010;11:503–13.

7. Aron AR, Robbins TW, Poldrack RA. Inhibition and the right inferior frontal cortex. Trends Cogn Sci. 2004;8:170–7.

8. Numssen O, Bzdok D, Hartwigsen G. Functional specialization within the inferior parietal lobe across cognitive domains. Elife. 2020;1–25.

9. Salgado S, Kaplitt MG. The nucleus accumbens: A comprehensive review. Stereotact Funct Neurosurg. 2015;93:75–93.

10. Cavanna AE, Trimble MR. The precuneus: A review of its functional anatomy and behavioural correlates. Brain. 2006;129:564–83.

11. Svoboda K, Li N. Neural mechanisms of movement planning: motor cortex and beyond. Curr Opin Neurobiol [Internet]. Elsevier Ltd; 2018;49:33–41. Available from: https://doi.org/10.1016/j.conb.2017.10.023

12. Ibanez A, Manes F. Contextual social cognition and the behavioral variant of frontotemporal dementia. Neurology. 2012;78:1354–62.

13. Bigler ED, Mortensen S, Neeley ES, Ozonoff S, Krasny L, Johnson M, et al. Superior temporal gyrus, language function, and autism. Dev Neuropsychol. 2007;31:217–38.

14. Wilterson AI, Nastase SA, Bio BJ, Guterstam A, Graziano MSA. Attention, awareness, and the right temporoparietal junction. Proc Natl Acad Sci U S A. 2021;118.

15. Morales M, Margolis EB. Ventral tegmental area: Cellular heterogeneity, connectivity and behaviour. Nat Rev Neurosci. 2017;18:73–85.
